# The Laccase-like Property of GHK-Cu and Its Applications in Colorimetric Sensing of Phenolic Compounds

**DOI:** 10.3390/bios16040217

**Published:** 2026-04-12

**Authors:** Jiang-Shan Chen, Huan Zhu, Tong-Qing Chai, Feng-Qing Yang

**Affiliations:** Department of Pharmaceutical Engineering, School of Chemistry and Chemical Engineering, Chongqing University, Chongqing 401331, China; 202518021108t@stu.cqu.edu.cn (J.-S.C.); 202318021131t@stu.cqu.edu.cn (H.Z.)

**Keywords:** laccase like, GHK-Cu, colorimetric sensing, phenolic compound, cotton-based sensor

## Abstract

Laccase plays an important role in the detection and degradation of phenolic compounds, but it is limited by its cost and stability. In this study, the laccase-like property of copper peptide (GHK-Cu) has been revealed. In terms of enzymatic reaction kinetics, GHK-Cu has a *V*_max_ of 1.735 × 10^−4^ mM·s^−1^ and a *K*_m_ of 0.061 mM, demonstrating good substrate affinity and excellent catalytic efficiency. Then, a colorimetry was developed for rapid detection of epinephrine (EP) and 2-aminophenol (2-AP). The linear response range of EP is 20–240 μM, with a limit of detection (LOD) of 9.5 μM. The linear response ranges of 2-AP are 14–100 μM (in ultrapure water) and 2–120 μM (in seawater), with LODs of 2.56 μM and 1.65 μM. In addition, combined with a smartphone platform, a cotton-based sensor has been developed for the detection of 2-AP in seawater. The linear response ranges are 0–0.2 mM and 0.2–1 mM, with LOD of 0.033 mM. The structure of GHK-Cu provides a reference for the development of novel laccase mimetic enzymes. The constructed colorimetry offers an option for the rapid detection of phenolic compounds, and the developed cotton-based sensor enabled rapid and portable detection of 2-AP.

## 1. Introduction

Phenolic compounds are a class of hydroxy-containing aromatic hydrocarbons that are widely distributed in nature, and can be divided into endogenous and exogenous phenols. Abnormal fluctuations in the concentration of endogenous phenolic compounds are closely associated with several diseases [1], but exogenous phenolic compounds are usually toxic organic pollutants, which may seriously harm the ecosystem and human health [2]. In modern industrial systems, phenolic compounds are widely used in the pharmaceutical, dye, pesticide, and paper-making industries [3,4,5]. These compounds can easily spread, seeping into water body and soil, posing a serious threat to the ecological environment [6]. Moreover, these phenolic compounds may enter the human body through various pathways, causing irreversible damage [7]. Therefore, developing low-cost, highly sensitive, and simple methods for detecting phenolic compounds is of great significance.

Commonly used methods for detecting phenolic compounds include chemical analysis, spectroscopic analysis, chromatographic analysis, and electrochemical analysis [8,9,10]. These methods have several advantages, such as being technologically mature and providing accurate results, but they also have some drawbacks like expensive equipment, complex sample pretreatment, and long analysis times. As a part of spectroscopic analysis, colorimetric detection provides advantages over traditional methods in terms of operational simplicity, low cost, and visual readout capability [11]. Therefore, developing new methods for detecting phenolic compounds based on colorimetric detection has gradually become a focus.

As a member of the multicopper oxidase family [12], laccase possesses several distinct characteristics, such as high enzymatic activity, long service life, and a broad substrate range. Laccase can not only efficiently catalyze the degradation of phenolic pollutants, but also catalyze several other types of compounds, such as aromatic amines (biphenyl amine), polycyclic aromatic hydrocarbons (anthracene, phenanthrene), synthetic dyes (Congo red, tartrazine), pesticides (chlorpyrifos), and antibiotics (tetracycline) [13,14,15]. And during the catalytic process, it produces only water as the reduction product, without generating harmful substances, such as H_2_O_2_. However, as a natural enzyme, laccase still faces issues like high cost and poor stability in practical applications. On the other hand, enzyme mimics generally have simpler structures and more stable chemical properties, which feature high catalytic efficiency, high selectivity, and low production costs, making them potential alternatives to natural enzymes [16]. In recent years, based on the structure of natural laccase, a series of copper-based nanomaterials with laccase-like activity have been extensively reported, such as Cu-DNA [17], Cu-amino acid [18], and Cu-protein coordination compound [19]. In addition, there are several other metal nanomaterials that exhibit laccase-like activity, such as platinum nanoparticles [20], manganese oxide [21], and rhodium single-atom nanozyme [22]. Although significant progress has been made in the preparation of different nanozymes with laccase-like activity, there remains considerable room for improvement in enhancing their enzymatic activity and designing laccase mimics with different structure. The copper peptide (GHK-Cu) is a compound formed by the combination of tripeptide glycyl-L-histidyl-L-lysine (GHK) with Cu^2+^ [23]. GHK-Cu has been found in human serum and plasma and is non-toxic [24,25]. In addition, GHK-Cu has antioxidant and anti-inflammatory effects, making it usable in the cosmetics industry [26]. It can also be used for skin healing and to induce fungi to increase laccase production [27,28].

**Scheme 1:**
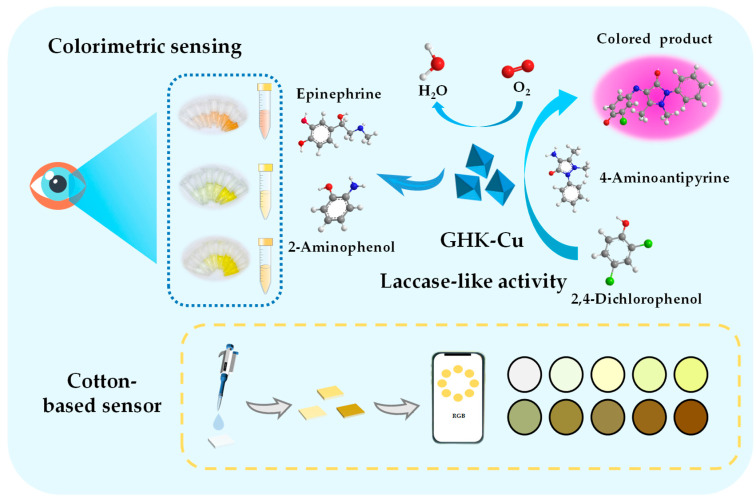
Schematic diagram of the laccase-like activity of GHK-Cu and colorimetric sensing application.

In this study, GHK-Cu was discovered for the first time to possess significant laccase-like activity, which can catalyze the oxidation of phenolic compounds through an electron transfer system based on the Cu(II)/Cu(I) redox cycle. This GHK-Cu has improved catalytic activity and stability over the natural laccase. Furthermore, GHK-Cu can be easily obtained commercially without the need for synthesis. Leveraging the exceptional laccase-like activity of GHK-Cu, a colorimetric method for the rapid detection of epinephrine (EP) and 2-aminophenol (2-AP) was developed, and has been successfully utilized in real sample analysis. In addition, a simple, portable cotton-based sensor integrated with a smartphone platform was developed for detecting 2-AP in seawater (Scheme 1).

## 2. Materials and Methods

### 2.1. Materials and Reagents

Copper Peptide (1:1) Acetate (GHK-Cu, ≥98%) was purchased from Shaanxi New Research Biosciences Co., Ltd. (Xi’an, China). Laccase was obtained from Beijing Solarbio Life Sciences Co., Ltd. (Beijing, China). Human serum albumin (HSA) was purchased from Beijing BioDee Biotechnology Co., Ltd. (Beijing, China). The 2-AP was purchased from Beijing Mreda Technology Co., Ltd. (Beijing, China). Sodium chloride (NaCl), anhydrous ethanol, potassium chloride (KCl), anhydrous sodium sulfate (Na_2_SO_4_), zinc sulfate heptahydrate (ZnSO_4_·7H_2_O), magnesium sulfate heptahydrate (MgSO_4_·7H_2_O), manganese sulfate monohydrate (MnSO_4_·H_2_O), nickel chloride hexahydrate (NiCl_2_·6H_2_O), lead nitrate (Pb(NO_3_)_2_), and lactose were obtained from Chengdu Kelong Chemical Co., Ltd. (Chengdu, China). Sodium acetate was obtained from Guangdong Guanghua Sci-Tech Co., Ltd. (Shantou, China). Isopropyl epinephrine hydrochloride, (±)-epinephrine hydrochloride, β-naphthol, and 2,4,6-trichlorophenol were purchased from Shanghai Aladdin Chemistry Co., Ltd. (Shanghai, China). 2,4-Dichlorophenol (2,4-DP), 2-morpholine ethysulfonic acid (MES), 4-hydroxyethylpiperazine ethylenesulfonic acid (HEPES), and bisphenol A (BPA) were purchased from Meryer (Shanghai) Chemical Technology Co., Ltd. (Shanghai, China). 4-Aminoantipyrine (4-AP), cadmium chloride hemi (pentahydrate) (CdCl_2_·5H_2_O), cobalt chloride hexahydrate (CoCl_2_·6H_2_O), fructose, water-soluble starch, and 2,6-dimethoxyphenol were obtained from Shanghai Macklin Biochemical Co., Ltd. (Shanghai, China). Epinephrine Hydrochloride Injection (Veterinary) was obtained from Shanghai Quanyu Biotechnology Co., Ltd. (Shanghai, China). Sodium dihydrogen phosphate (NaH_2_PO_4_), maltose, 2-chlorophenol, and 1,3-resorcinol were purchased from Shanghai Titan Scientific Co., Ltd. (Shanghai, China). Bovine serum albumin (BSA) was purchased from Sigma-Aldrich (Shanghai) Trading Co., Ltd. (Shanghai, China). D(+)-Glucose was obtained from Shanghai Yuanye Bio-Technology Co., Ltd. (Shanghai, China). Calcium chloride (CaCl_2_) was obtained from Tianjin Damao Chemical Reagent Partnership Enterprise (Limited Partnership) (Tianjin, China). Phenol was purchased from Chongqing Chuandong Chemical (Group) Co., Ltd. (Chongqing, China). Face towel was obtained from Hefei Suruanruan Brand Management Co., Ltd. (Hefei, China). All chemicals were used as received without further purification. Ultrapure water utilized throughout all experiments was obtained from a water-purification apparatus ATSelem 1820A, Antesheng Environmental Protection Equipment Co., Ltd. (Chongqing, China).

### 2.2. Apparatus and Measurements

Scanning electron microscopy (SEM) images and an element distribution map were obtained using Quanta 650 field-emission scanning electron microscope (Thermo Fisher Scientific, Waltham, MA, USA). The Fourier transform infrared (FT-IR) spectra were recorded using a Nicolet iS50 spectrometer (Thermo Fisher Scientific, Waltham, MA, USA, scanning wavelength range of 400–4000 cm^−1^). X-ray diffraction (XRD) was conducted on an X’pert Powder diffractometer (Malvern Panalytical Ltd., Almelo, OV, The Netherlands) with Cu Kα radiation. X-ray photoelectron spectroscopy (XPS) was obtained using a PHI5000 Versaprobe system using monochromatic Al Ka radiation (1486.6 eV) (Thermo Fisher Scientific Ltd., Hemel Hempstead, HRT, UK, the standard peak position of carbon is 284.80 eV). The thermal stability of GHK-Cu was analyzed by TGA2 (Mettler-Toledo AG, Analytical, Greifensee, ZH, Switzerland), heated from 30 °C to 800 °C at a heating rate of 10 °C∙min^−1^ under air gas flow.

### 2.3. Exploration of the Laccase-like Activity of GHK-Cu and Laccase

In the presence of oxygen, laccase can catalyze the oxidation of phenolic compounds (2,4-DP) and coupling with a color reagent (4-AP) to produce a rose-red colored product (the maximum absorption wavelength at 510 nm). Therefore, the catalytic activity of GHK-Cu was determined according to the chromogenic reaction of phenolic compounds with 4-AP. Specifically, 2,4-DP aqueous solution (100 μL, 1 mM), 4-AP aqueous solution (100 μL, 1 mM), and GHK-Cu aqueous solution (100 μL, 2 mg/mL) were added to ultrapure water (200 μL) in 500 μL centrifuge tube and reacted at 70 °C for 10 min. Subsequently, the absorption spectrum of the reaction solution was measured using a UV-Vis spectrophotometer (Shanghai Metash Instruments, Shanghai, China), and the absorbance at 510 nm was recorded. Under similar conditions, the catalytic activity of natural laccase was examined. The concentration of the laccase aqueous solution was 10 mg/mL, and the reaction time was 30 min, and the reaction solution was centrifuged for 2 min (4000 rpm). Then, the experimental conditions, such as the type and pH of buffer, the reaction temperature, the reaction time, and the initial mass concentration of catalyst, were investigated to obtain the optimized catalytic conditions for GHK-Cu and laccase.

### 2.4. Study on the Enzyme Catalyzed Reaction Kinetics

To investigate the reaction kinetics of GHK-Cu, the absorbance at 510 nm of the reaction mixture was monitored under optimized reaction conditions, but varying the initial concentration of 2,4-DP (0.1–1.5 mM) in the reaction system. In detail, GHK-Cu solution (100 μL, 2 mg/mL), 2,4-DP (100 μL), and 4-AP (100 μL, 1 mM) were added to MES (200 μL, 10 mM, pH 7) in a 500 μL centrifuge tube and reacted at 80 °C for 12 min. The reaction kinetics parameters (*K*_m_ and *V*_max_) were calculated using the Michaelis–Menten equation (Equation (1)) and Lineweaver Burk equation (Equation (2)) [29].

(1) $$V=\frac{V_{\max}[S]}{K_{m}+[S]}$$

(2) $$\frac{1}{V}=\frac{K_{m}}{\;V_{\max}[S]}+\frac{1}{V_{\max}}$$

where *V* represents the initial reaction rate, *V*_max_ denotes the maximum reaction rate, *K*_m_ is the Michaelis–Menten constant, and [*S*] is the substrate concentration.

The catalytic efficiency with respect to mass concentration (*k*_cat_/*K*_m_) was calculated.

(3) $$k_{\mathrm{cat}}/K_{m}=\frac{V_{\max}}{K_{m}[E_{0}]}$$

where *k*_cat_ is catalytic constant, and [*E*_0_] is initial mass concentration of the catalyst.

### 2.5. Assessment of Catalytic Stability

To evaluate the effect of ionic strength on the catalytic reaction process of GHK-Cu, NaCl was introduced into the reaction system. Specifically, different amounts of NaCl (NaCl dissolved into MES, and the final concentrations are 0–400 mM) were added to the reaction system and measured according to the method described in Section 2.4, and then the relative activity was immediately determined with the comparison of the activity at 0 mM of NaCl. Under the optimized catalytic conditions for laccase, its activity was assayed under the same experimental conditions and compared with that of GHK-Cu.

To investigate the effect of organic solvents on the GHK-Cu catalysis, ethanol solution of varying volume fractions (0, 10%, 20%, 30%, 40%) were introduced into the reaction system, and their effects were compared with the activity of a blank control group. And the results of GHK-Cu and laccase were compared.

To evaluate the effect of potential interfering metal ions in the reaction process, solutions containing different ions (K^+^, Na^+^, Ca^2+^, Zn^2+^, Mg^2+^, Mn^2+^, Ni^2+^, Pb^2+^, Cd^2+^, Co^2+^, final concentration is 10 mM) in MES was prepared and to replace the original MES solution in the reaction system. The absorbance was then measured and compared with that of a blank control group.

To assess the effect of organic substances on the catalytic process, various types of organic compounds, including human serum albumin (HSA), bovine serum albumin (BSA), D-glucose (D-Glu), fructose (Fru), lactose (Lac), maltose (Mal), and amylum (Amy), which were dissolved into MES and added to the reaction system. Following the reaction, the absorbance of the solution was measured and compared to that of a blank control group. It is noted that the final concentrations of HSA and BSA were 1 mg/mL, and other organic compounds were maintained at 10 mM.

In addition, to evaluate the storage stability of GHK-Cu, it was dissolved in MES (10 mM, pH 7) and stored at 4 °C. The activity was measured at 24 h intervals and compared to its initial activity (day one).

### 2.6. Degradation of Phenolic Compounds

To investigate the catalytic degradation capability of GHK-Cu toward different phenolic compounds, 2,4-DP was replaced with other phenolic substrates, including 2-Aminophenol (2-AP), β-Naphthol (β-NP), Phenol, 2-Chlorophenol (2-CP), 2,4,6-Trichlorophenol (2,4,6-TP), m-Cresol, Resorcinol (R), 2,6-Dimethoxyphenol (2,6-DOP), Bisphenol A (BPA), Isoprenaline (ISO) and Epinephrine (EP). And the absorbance was measured and compared with that of the 2,4-DP. Notably, ISO, EP, and 2-AP can undergo a color change upon catalytic oxidation by GHK-Cu, thus eliminating the need for adding the developer 4-AP to the reaction mixture.

Afterwards, the degradation of 2,4-DP (1 mM) in simulated sewage by GHK-Cu (2 mg/mL) was investigated using a first-order kinetic model, and its potential for practical applications was evaluated. The first-order rate constant was determined using Equation (4) [30].

(4) $$\ln\frac{C_{0}}{C_{t}}=k_{1}t$$

where *C*_0_ denotes the initial concentration of 2,4-DP, *C*_t_ its concentration at time *t* (min), and *k*_1_ is the first-order reaction rate constant.

### 2.7. Establishment of Colorimetric Detection Method

Based on the remarkable laccase-like activity of GHK-Cu, a colorimetric method was developed for the detection of 2-AP and EP. Specifically, varying concentrations of phenolic substrates (100 μL) were introduced into MES (300 μL, 10 mM, pH 7), followed by the addition of GHK-Cu solution (100 μL, 2 mg/mL). The reaction was carried out at 80 °C for 12 min, after which the absorbance of the resulting mixture was immediately measured. A linear calibration curve was constructed through plotting the absorbance values against the corresponding substrate concentrations to determine the linear detection range. The limit of detection (LOD) was calculated based on the formula 3*σ*/*b*, where *σ* denotes the standard deviation of the blank signal and *b* is the slope of the calibration curve. It is noted that 2-AP was dissolved in seawater, and the calibration curve of 2-AP in seawater was re-established using the method described in this section, for the analysis of 2-AP in seawater and the maximum absorption wavelength is 434 nm for 2-AP and 479 nm for EP.

### 2.8. Real Sample Analysis

The established method was applied to quantitatively detect EP in veterinary Epinephrine Hydrochloride Injection. In brief, the injection was diluted 1:4 with ultrapure water to ensure that its concentration fell within the linear range of the detection method, and the tests were conducted according to the experimental conditions described in Section 2.4. The accuracy was evaluated through comparing the measured values with the labeled content and calculating relative standard deviation (RSD). To assess repeatability, three separate vials of the injection were tested consecutively under the same conditions, with each vial analyzed in triplicate.

The developed colorimetric method was applied in detecting 2-AP and EP in real environmental water samples. Spike recovery tests for 2-AP were performed using tap water, lake water (from Jin Lake on the Huxi Campus of Chongqing University), and seawater (from the Bohai Sea). In brief, varying concentrations of 2-AP (diluted in the corresponding water matrix, 100 μL) were added to MES (300 μL), followed by the introduction of GHK-Cu solution (100 μL). The mixture was incubated at 80 °C for 12 min and analyzed using the colorimetric method described in Section 2.4. The accuracy of the method was evaluated by spike recovery rates and RSD. All water samples were filtered through a 0.22 μM membrane to remove insoluble impurities. To minimize interference from chloride ions, both tap water and lake water were diluted 100-fold with ultrapure water. Method repeatability was assessed via three independent replicate measurements under identical conditions.

### 2.9. Construction of Portable Cotton-Based Sensor

A simple and portable colorimetric strip was developed using cotton towel and coupled with a smartphone, enabling real-time detection of 2-AP in the Bohai Sea water. Specifically, cotton towel was cut into 1 cm × 1 cm sections, immersed in MES (10 mM, pH 7) containing GHK-Cu (0.5 mg∙mL^−1^) for 5 min, and then vacuum-dried at 60 °C for 5 min. Varying concentrations of 2-AP (dissolved in seawater) were applied to the test strips, then reacted for 12 min at 80 °C. To reduce external environmental interference, the reacted cotton-based sensors are transferred to a small photo studio for image capture. The small photo studio has a circular opening at the top, and using the rear camera of the smartphone to shoot vertically from the top opening. (The smartphone remains at default settings). The *R*, *G*, and *B* values are obtained by processing the image through the “Color Picker” APP. These values were converted using Equation (5) to generate a concentration-response plot. The concentration of 2-AP in the actual samples was quantified by the standard addition method.

(5) $$\mathrm{Value}=\frac{\left(R-B\right)}{G}$$

## 3. Results and Discussion

### 3.1. Characterization of GHK-Cu

**Figure 1:**
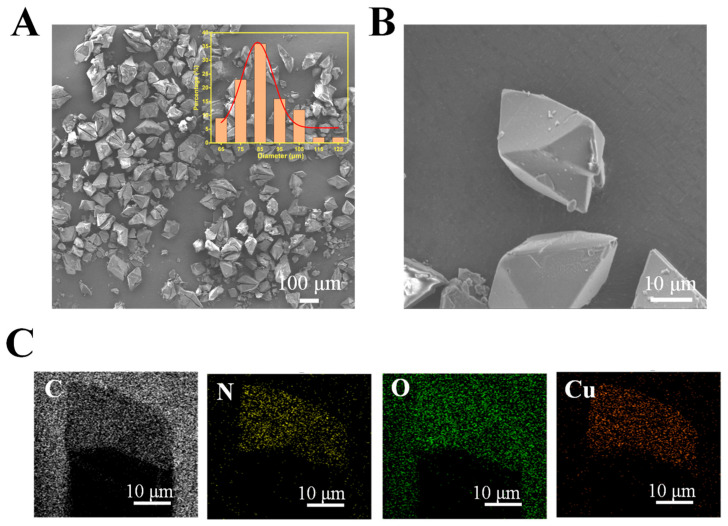
SEM images of GHK-Cu (**A**,**B**) (insets of the particle size distribution diagram fitted by Gaussian curve). EDX mapping image of GHK-Cu (**C**).

**Figure 2:**
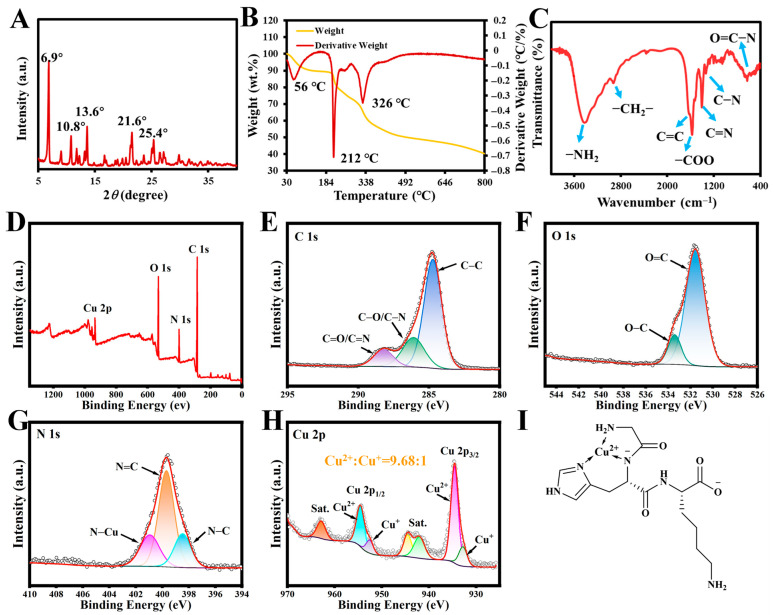
XRD pattern (**A**), TGA pattern (**B**), and FT-IR spectra (**C**) of GHK-Cu. The XPS spectra of GHK-Cu: the full-scan survey spectrum (**D**), C 1s spectrum (**E**), O 1s spectrum (**F**), N 1s spectrum (**G**), Cu 2p spectrum (**H**). Black circles represent the original data, the red line is the fitting curve, and the fitting peaks filled with different colors correspond to different structural information. Molecular structure of GHK-Cu (**I**).

As shown in Figure 1A,B, GHK-Cu has an octahedral structure, with an average particle size of approximately 85 μM based on the particle size distribution analysis. Energy dispersive X-ray spectroscopy (EDX) elemental mapping of Figure 1B further reveals the uniform distribution of C, N, O, and Cu in the GHK-Cu material (Figure 1C). The crystal structure of GHK-Cu was characterized by XRD. As shown in Figure 2A, GHK-Cu displays well-defined diffraction peaks at 2*θ* values of 6.9°, 10.8°, 13.6°, 21.6°, and 25.4°, indicating high crystallinity. Subsequently, thermogravimetric analysis (TGA) was performed to evaluate the thermal stability of GHK-Cu. As depicted in Figure 2B, major mass loss events occurred at 56 °C, 212 °C, and 326 °C. The initial loss at 56 °C is attributed to the evaporation of surface-adsorbed water, while the subsequent losses at 212 °C and 326 °C are likely associated with the decomposition of residual acetic acid and the peptide structure of GHK-Cu, respectively. The molecular structure of GHK-Cu was further characterized by FT-IR spectroscopy. As shown in Figure 2C, the peak at 3415 cm^−1^ corresponds to the N–H stretching vibration, while the absorption at 2925 cm^−1^ is assigned to the –CH_2_– stretching vibration. The peaks at 1618 cm^−1^ and 1571 cm^−1^ are attributed to the C=C stretch and the asymmetric vibration of the carboxylate group (–COO^−^), respectively. Additionally, absorptions at 1405 cm^−1^, 1374 cm^−1^, and 624 cm^−1^ are associated with the C=N stretch, C–N stretch, and the in-plane bending of C–N in the amide moiety (O=C–N), respectively.

The surface elemental composition and chemical states of GHK-Cu were further investigated using XPS. As shown in Figure 2D, the full survey spectrum confirms the presence of C, O, N, and Cu, which is in consistent with the EDX elemental mapping results in Figure 1C. In the C 1s spectrum (Figure 2E), peaks observed at 284.80 eV, 286.48 eV, and 288.18 eV are assigned to the C–C, C–O/C–N, and C=O/C=N species, respectively. The O 1s spectrum (Figure 2F) displays two peaks at 531.58 eV and 533.38 eV, corresponding to the O=C and O–C functional groups, respectively. Figure 2G shows the N 1s spectrum, where peaks at 399.66 eV, 401.48 eV, and 398.08 eV can be attributed to N=C, N–Cu coordination bonds, and N–C species, respectively. Notably, the presence of N–Cu coordination bonds is in consistent with previously reported laccase mimics, exhibiting similar binding energies [31]. Additionally, the Cu 2p spectrum of GHK-Cu is presented in Figure 2H, showing characteristic peaks at 934.58 eV and 954.38 eV, corresponding to the Cu 2p_3/2_ and Cu 2p_1/2_ orbitals, respectively. Deconvolution of the Cu 2p_3/2_ signal reveals peaks at 934.57 eV and 932.20 eV, which are assigned to the Cu^2+^ and Cu^1+^, respectively. Similarly, the Cu 2p_1/2_ signal was fitted with peaks at 954.40 eV (Cu^2+^) and 952.22 eV (Cu^1+^), confirming the coexistence of Cu^2+^ and Cu^1+^ in GHK-Cu with a ratio of about 9.68:1. These Cu species are critical for the laccase-like activity of GHK-Cu.

Based on the FT-IR and XPS results, the structure of GHK-Cu was proposed and illustrated in Figure 2I. Cu^2+^ is coordinated in a tridentate fashion within the peptide framework, and the presence of the Cu–N coordination bond is confirmed by XPS analysis. The structure is consistent with previously reported results [32].

### 3.2. Evaluation of the Laccase-like Catalytic Activity of GHK-Cu

**Figure 3:**
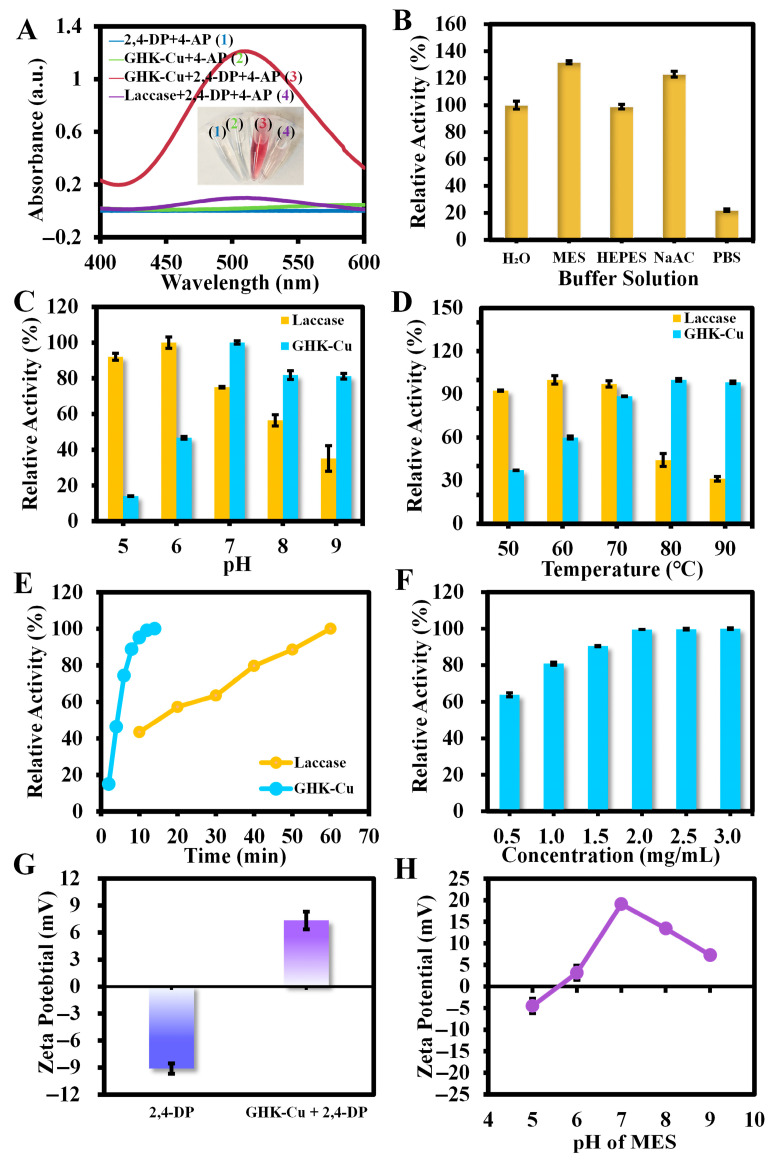
UV-Vis absorption spectra of different reaction solutions containing 4-AP (**A**). Effects of the type (**B**) and pH (**C**) of buffer on the laccase-like activity of GHK-Cu. Effects of reaction temperature (**D**), reaction time (**E**), and the initial mass concentration of GHK-Cu (**F**) on the laccase-like activity of GHK-Cu. The Zeta potential of the individual 2,4-DP solution and the 2,4-DP solution containing GHK-Cu (**G**). Zeta potential of the reaction solution with MES of different pH (**H**). The error bars represent the standard deviation of three independent measurements.

The laccase-like catalytic activity of GHK-Cu was evaluated by the benchmarked chromogenic reaction of 2,4-DP with 4-AP. As shown in Figure 3A, a pronounced increase in absorbance was observed only in the presence of either GHK-Cu or laccase. Notably, the absorbance change at 510 nm induced by GHK-Cu was substantially greater than that of laccase. The development of a rose-red color in the corresponding samples (inset) further supports the superior laccase-like activity of GHK-Cu as compared to the natural enzyme. As illustrated in Figure 3B–F, the catalytic activity of GHK-Cu is governed by buffer type, pH, reaction temperature, reaction time, and catalyst dosage. The optimized reaction conditions of GHK-Cu were established to be using MES (10 mM, pH 7) as buffer, a temperature of 80 °C, a reaction time of 12 min, and a GHK-Cu concentration of 2 mg·mL^–1^.

The optimized reaction conditions for natural laccase are as follows: MES (10 mM, pH 6), reaction temperature (60 °C), reaction time (60 min), concentration (10 mg·mL^–1^). It can be observed that, compared to natural laccase, GHK-Cu is more heat-resistant and requires a shorter reaction time under their respective optimized reaction conditions (Figure 3D,E). Furthermore, it is evident that the catalytic activity of GHK-Cu is easily influenced by the pH of MES. As shown in Figure 3G, the zeta potential of the 2,4-DP solution alone is negative, whereas it becomes positive after the addition of GHK-Cu. This indicates that the substrate binds with GHK-Cu through electrostatic interactions. As the pH of MES continues to rise, the zeta potential of the reaction solution shows a trend of first increasing and then decreasing, reaching its maximum at a pH of 7 (Figure 3H). This is similar to the trend shown in Figure 3C. This indicates that pH can influence the electrostatic interactions between the substrate and GHK-Cu, thereby affecting its catalytic activity.

### 3.3. The Enzymatic Kinetics and Degradation Kinetics of GHK-Cu

**Figure 4:**
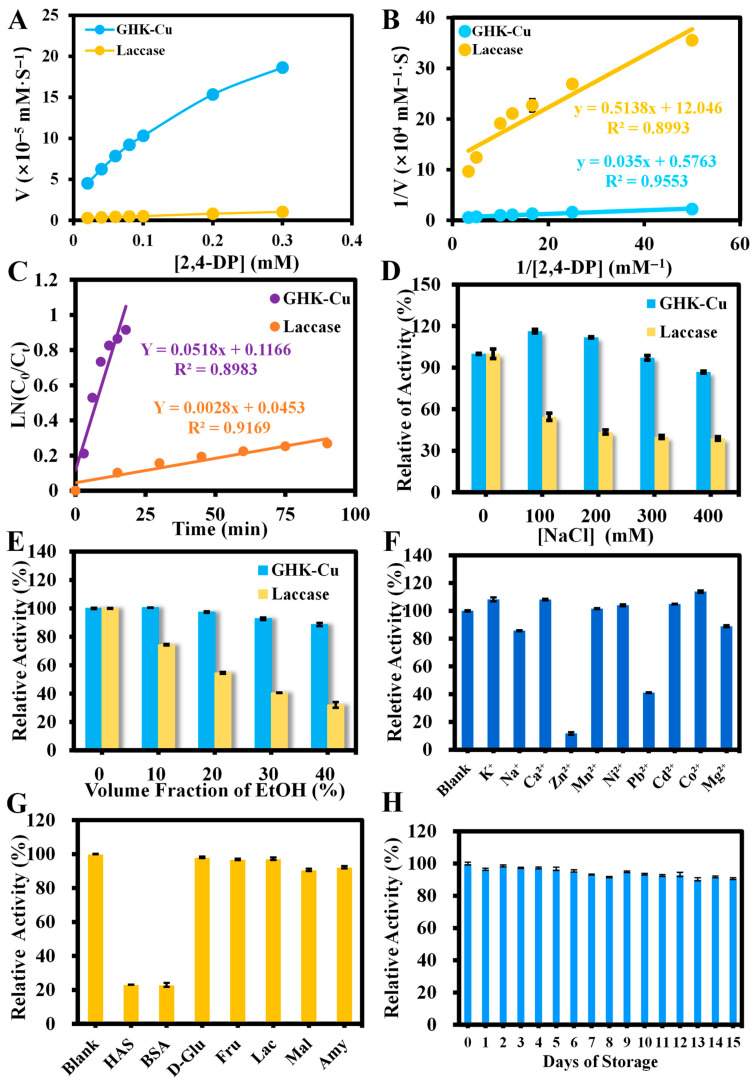
Graph of 2,4-DP concentration and catalytic reaction rate for GHK-Cu/laccase (**A**). Double reciprocal plots of the enzyme kinetics for GHK-Cu/laccase (**B**). Degradation kinetics diagram of GHK-Cu/laccase (**C**). The effects of ionic strength (**D**) and ethanol (**E**) on the catalytic activity of GHK-Cu/laccase. Effects of metal ions (**F**) and organic substances (**G**) on the catalytic activity of GHK-Cu. The effects of storage time on the catalytic activity of GHK-Cu (**H**). The error bars represent the standard deviation of three independent measurements.

To further understand the catalytic activity of GHK, the enzymatic kinetics of GHK and natural laccase were measured (Figure 4A,B), and the fitted kinetic parameters are listed in Table 1. The experimental results show that GHK-Cu has a maximum reaction rate (*V*_max_) of 1.735 × 10^−4^ mM·s^−1^, a Michaelis constant (*K*_m_) of 0.061 mM, and a catalytic efficiency (*k*_cat_/*K*_m_) of 14.3 × 10^–4^ (g·L^–1^)^–1^∙s^–1^). Compared with the results of laccase (*V*_max_ = 0.083 × 10^−4^ mM·s^−1^, *K*_m_ = 0.043 mM; *k*_cat_/*K*_m_ = 0.195 × 10^–4^ (g·L^–1^)^–1^∙s^–1^, GHK-Cu has a slightly higher *K*_m_, while its *V*_max_ and *k*_cat_/*K*_m_ values are significantly higher than those of laccase. Compared with the other materials listed in Table 1, GHK-Cu has a lower *K*_m_, demonstrating good affinity to the substrate. At the same time, GHK-Cu has higher *V*_max_ and *k*_cat_/*K*_m_, indicating relatively good catalytic activity. These results demonstrate the excellent laccase-like activity of GHK-Cu. In addition, as shown in Figure 4C, the calculated kinetic constant for GHK-Cu in 2,4-DP degradation is 0.0518 min^−1^, which is higher than that of laccase (0.0028 min^−1^). Therefore, GHK-Cu exhibited excellent catalytic performance and held potential for the low-cost and efficient catalytic degradation of phenolic compounds.

### 3.4. Investigation of the Catalytic Stability of GHK-Cu

In order to investigate the catalytic stability of GHK-Cu and further assess its practical application value, the effects of ionic strength, organic solvents, metal ions, organic compounds, and storage time on the catalytic activity of GHK-Cu were studied.

The changes in the activities of GHK-Cu and natural laccase at different NaCl concentrations (0–400 mM) were measured. As shown in Figure 4D, with the continuous increase in NaCl concentration, the relative activity of GHK-Cu first gradually increases (100–111.8%), and then slightly decreases (111.8–86.8%), whereas the relative activity of laccase continuously declines (100–38.8%). At low ionic strength, the relative activity of GHK-Cu shows an enhanced phenomenon, which may be due to O_2_ oxidizing chloride ions into reactive chlorine, which can further oxidize the substrate 2,4-DP [29]. At high ionic strengths, the elevated concentration of NaCl significantly increases the ionic strength of the solution, leading to an electrostatic shielding effect that weakens the electrostatic interactions between GHK-Cu and 2,4-DP, thereby reducing the catalytic activity. The decrease in laccase-like activity may be due to the salting-out effects and inactivation by chloride ions. Then, with the continuous increase in the volume fraction of the added ethanol solution, the relative activity of GHK-Cu showed a slight decrease (100–88.6%), whereas the relative activity of laccase showed a significant decrease (100–32.0%) (Figure 4E). Even with the addition of 40% (*v*/*v*) ethanol solution, GHK-Cu can still maintain 88.6% of its relative activity, while laccase can retain only 32.0%, indicating that GHK-Cu has better stability in ethanol solution. In addition, the effects of metal ions (K^+^, Na^+^, Ca^2+^, Zn^2+^, Mg^2+^, Mn^2+^, Ni^2+^, Pb^2+^, Cd^2+^, and Co^2+^) on the catalytic activity of GHK-Cu were examined by introducing them into the reaction solution. As it can be seen from Figure 4F, for most metal ions, GHK-Cu exhibits good interference resistant ability, with relative activity remaining above 85%. Notably, in solutions containing K^+^, Ca^2+^, and Co^2+^, GHK-Cu shows a slight increase in relative activity. This may be due to the use of their chloride salts when preparing the solutions, resulting in a small amount of chloride ions in the reaction solution, which can enhance the catalytic activity of GHK-Cu. When Zn^2+^ or Pb^2+^ are present in the reaction solution, the relative activity of GHK-Cu significantly decreases (by 11.7% and 41.0%, respectively). The Pb^2+^ can affect the catalytic activity of GHK-Cu may be due to the coordination of Pb^2+^ with the −COO^−^ and −NH_2_ groups in the GHK-Cu structure, which may disrupt its structure. Furthermore, it is speculated that Zn^2+^ may form a bidentate complex with the histidine residues in the structure of GHK-Cu, greatly disrupting its structure and consequently causing a substantial decrease in its catalytic activity [45].

Moreover, the effects of different organic compounds on the catalytic activity of GHK-Cu were investigated. As shown in Figure 4G, for most of the tested groups, the relative activity of GHK-Cu did not change significantly, indicating that GHK-Cu possesses good interference resistant ability against organic compounds. However, the relative activity of GHK-Cu decreased substantially when reaction solutions containing HAS or BSA. This is likely because serum proteins non-specifically adsorb to GHK-Cu through electrostatic interactions, masking the active sites, and hindering the binding of GHK-Cu to the substrate. In addition, the effect of storage time on the activity of GHK-Cu was investigated through measuring the activity of GHK-Cu preserved for different durations (up to 15 d). As shown in Figure 4H, after 15 days of storage at 4 °C, GHK-Cu retained 90.7% of its relative activity, indicating that GHK-Cu can maintain its laccase-like activity over an extended period, demonstrating good storage stability.

In short, GHK-Cu can maintain good catalytic activity under different ionic strengths, ethanol concentrations, metal ions, and organic compounds, and it can be stored for a long time. These features are meaningful for the long term, stable degradation of phenolic compounds.

### 3.5. The Catalytic Mechanism of GHK-Cu

**Figure 5:**
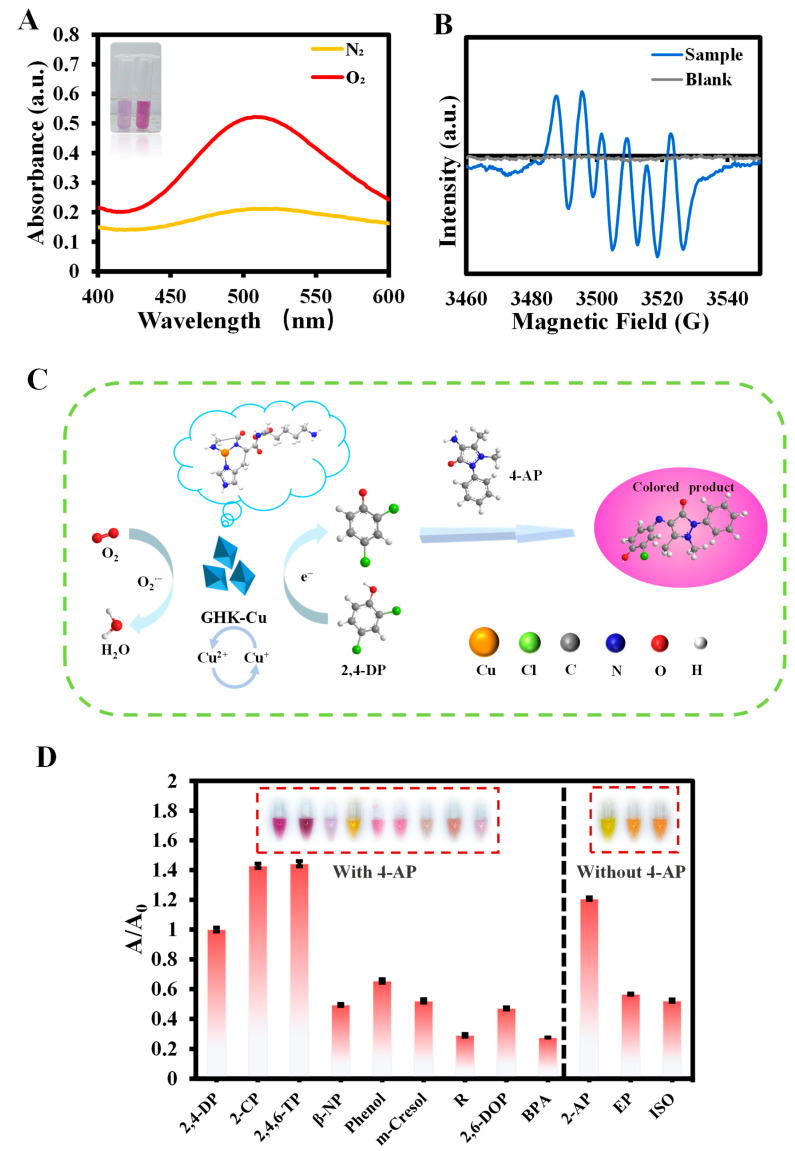
The results of N_2_/O_2_ gas saturation experiments (**A**). The results of EPR experiments for GHK-Cu (**B**). Possible mechanism of GHK-Cu catalyzed oxidation of 2,4-DP (**C**). The relative catalytic activities of GHK-Cu towards different phenolic compounds (using the absorbance of the 2,4-DP group as a reference) (**D**). Error bars represent the standard deviation of three repeated experiments.

To investigate the catalytic mechanism of GHK-Cu, the role of dissolved oxygen in the reaction solution was explored through N_2_/O_2_ gas saturation experiments. As illustrated in Figure 5A, the experimental group continuously supplied with O_2_ exhibited a markedly stronger characteristic absorption peak at 510 nm compared to N_2_ group, and the reaction solution was also darker in color. This indicates that the dissolved oxygen plays an important role in the catalytic reaction process. Furthermore, EPR experiments were conducted to reveal the active species during the catalytic reaction of GHK-Cu, and the result is shown in Figure 5B. It can be seen that six characteristic peaks appear in the spectrum, of which four peaks have relatively high intensity. This is a characteristic feature of the spectrum of superoxide anions (O_2_**^•^**^−^). This elucidates that during the catalytic reaction, O_2_ is converted into O_2_**^•−^** and acts as an active intermediate. Therefore, based on the mechanism of laccase catalyzed phenolic compounds, the structure of GHK-Cu, and EPR experiments, a possible mechanism for GHK-Cu catalyzed oxidation of 2,4-DP can be proposed. As shown in Figure 5C, 2,4-DP binds with GHK-Cu and is oxidized by GHK-Cu, losing one electron to form a phenoxy radical intermediate. The intermediate can couple with 4-AP to form a rose-red product, causing changes in the color of the reaction solution and its characteristic absorption peak. At the same time, Cu(II) in GHK-Cu accepts electrons from 2,4-DP, converting Cu(II) to Cu(I). Then, O_2_ immediately binds to Cu(I), transferring electrons to O_2_ and turning it into O_2_^•−^, and O_2_^•−^ with H^+^ reacts to form H_2_O, while Cu(I) is re-oxidized to Cu(II), completing the redox cycle of the copper ion. The catalytic cycle of GHK-Cu is similar to previously reported laccase mimics [30].

### 3.6. Study on the Catalytic Degradation of Various Phenolic Compounds by GHK-Cu

The phenolic pollutants 2,4-DP, 2-aminophenol (2-AP), β-naphthol (β-NP), phenol, 2-chlorophenol (2-CP), 2,4,6-trichlorophenol (2,4,6-TCP), m-cresol, resorcinol (R), 2,6-dimethoxyphenol (2,6-DOP), bisphenol A (BPA), as well as epinephrine (EP) and isoprenaline (ISO), to investigate the degradation capability of GHK-Cu towards different phenolic compounds. As shown in Figure 5D, GHK-Cu exhibits catalytic degradation capability toward various phenolic compounds, all of which produced colored products (as shown in the insets). Notably, for 2-AP, 2-CP, and 2,4,6-TCP, GHK-Cu demonstrated a stronger degradation ability than for 2,4-DP. These experimental results indicate that GHK-Cu can catalytically degrade a wide range of phenolic compounds and exhibit considerable potential for catalytic degradation of various phenolic compounds in practical applications.

### 3.7. Detection of EP

Epinephrine (EP), a catecholamine, is a stress marker in mammals. It is released by the adrenal medulla and functions to regulate blood pressure, cause vasoconstriction, increase heart rate, and act as a bronchodilator for asthma [46]. EP plays an important role in maintaining cardiovascular health, regulating blood sugar levels, and modulating the body’s response to various stimuli. Clinically, EP is used as a medication to treat a variety of conditions, including anaphylactic shock, cardiac arrest, and severe asthma attacks [39]. Therefore, it is of great significance to develop a fast, accurate, and low-cost method for detecting EP.

**Figure 6:**
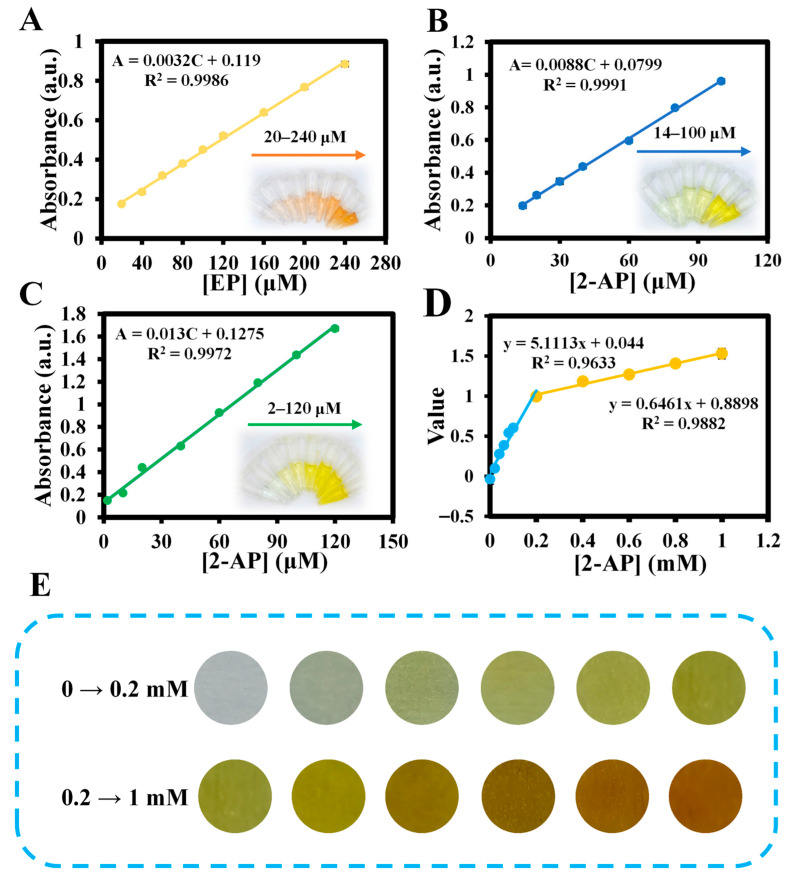
The relationship between EP concentration (**A**) and the corresponding absorption intensity at 479 nm. The relationship between 2-AP concentration in ultrapure and the corresponding absorption intensity at 434 nm (**B**). The relationship between 2-AP concentration in seawater and the corresponding absorption intensity at 434 nm (**C**). The relationship between 2,4-DP concentration and the calculated values (**D**). The color of the cotton-based sensor at different 2-AP concentrations (**E**). Error bars represent the standard deviation of three repeated experiments.

As manifested in Figure 6A, the calibration curve for EP has good linearity (*R*^2^ > 0.99) over the range of 20–240 μM, with a LOD of 9.5 μM. To further evaluate the established method, it was compared with several previously reported methods for detecting EP, as presented in Table 2. It can be seen that, compared with previously reported detection methods, the established EP colorimetric detection method has a wide detection range and a relatively low LOD. The established EP colorimetric detection method was applied to the actual sample testing of hydrochloric epinephrine injection (veterinary), and the detection results are shown in Table 3. The measured values of Epinephrine Hydrochloride Injection (veterinary) compared to the labeled values ranged from 105.6–108.5%, with an RSD between 0.9–1.4% (*n* = 3). Therefore, the established colorimetric detection method for EP can be applied to the accurate detection of hydrochloric epinephrine injection (veterinary) samples. This study provides a potential method for low-cost, rapid, and accurate detection of hydrochloric epinephrine injection (veterinary).

### 3.8. Detection of 2-AP

The 2-AP is an important chemical intermediate, widely used in industries, such as dyes and pharmaceuticals [55]. However, prolonged exposure to 2-AP may cause skin itching, allergic reactions, and is more likely to damage DNA [56,57]. Therefore, it is necessary to develop low-cost, rapid, and accurate methods for detecting 2-AP.

As shown in Figure 5D, GHK-Cu exhibits excellent catalytic degradation capability towards 2-AP, and there is a characteristic absorption peak at 434 nm. Based on this characteristic, a colorimetric method for detecting 2-AP was established. It can be seen from Figure 6B,C, the calibration curves of 2-AP were determined in ultrapure water and seawater, respectively. As the concentration of 2-AP continues to increase, its absorption peak intensity at 434 nm gradually strengthens. Whether in ultrapure water or seawater, the obtained calibration curves show good linearity (*R*^2^ > 0.99). The detection range in ultrapure water is 14–100 μM, with a LOD of 2.56 μM; in seawater, the detection range is 2–120 μM, with a LOD of 1.65 μM. Compared with previously reported methods for detecting 2-AP, the colorimetric detection method established in this study offers a wider detection range and a lower LOD (Table 4). At the same time, it can be seen from the illustration that as the concentration of 2-AP continues to increase, the color of the reaction solution becomes more pronounced, making it possible to detect 2-AP through visual color comparison. In order to further evaluate the practical application value of the established colorimetric detection method for 2-AP, it was applied to the spiked testing of actual samples (tap, lake, and Bohai Sea water). As shown in Table 5, in tap water samples, the recovery of 2-AP ranged from 102.6% to 104.5%, with RSD of 0.9–2.1% (*n* = 3); in Jin Lake water samples from Chongqing University, the recovery of 2-AP ranged from 93.5% to 104.0%, with RSD of 0.4–2.0% (*n* = 3); in Bohai Sea water samples, the recovery of 2-AP ranged from 102.6% to 104.5%, with RSD of 1.1–4.1% (*n* = 3). Therefore, the established colorimetric method for detecting 2-AP can be applied for accurate determination of 2-AP in real environmental water samples.

### 3.9. Portable Cotton-Based Sensor for Rapid Detection of 2-AP in Seawater

Compared to traditional paper-based sensors, cotton-based sensors that use cotton fibers as the support can better load catalysts and absorb reagents, which helps to promote the catalytic reaction [63]. In this study, a cotton-based sensor loaded with GHK-Cu was developed and combined with a smartphone platform for the rapid detection of 2-AP in Bohai Sea water. The R, G, and B values obtained from the software were calculated according to Equation (5), and the resulting values were plotted against the 2-AP concentration, as shown in Figure 6D. The detection range of 2-AP is 0–0.2 mM and 0.2–1.0 mM; the linear regression equations are *y* = 5.1113*x* + 0.044 (*R*^2^ = 0.9633) and *y* = 0.6461*x* + 0.8898 (*R*^2^ = 0.9882), respectively; LOD is 0.033 mM. As can be seen from Figure 6E, as the concentration of 2-AP increases, the color of the cotton-based sensor gradually darkens. Based on the obtained calibration curves, the spiking recovery method was used to detect 2-AP in Bohai Sea water, and the results are shown in Table 5. The recovery of 2-AP is in the range of 95.6–101.4%, with an RSD of 0.8–3.1%. Therefore, a portable cotton-based sensor based on GHK-Cu has been successfully developed and can achieve rapid detection of 2-AP in sea water.

## 4. Conclusions

This study is the first report to discover that the metal–peptide complex GHK-Cu exhibits significant laccase-like catalytic activity. Compared with natural laccase, GHK-Cu exhibits excellent substrate affinity and superior catalytic activity, as well as outstanding stability under different pH levels, ionic strengths, and storage conditions. GHK-Cu catalyzes the oxidation of substrates through electron transfer associated with the Cu(II)/Cu(I) redox cycle. Based on the excellent catalytic activity and stability of GHK-Cu, a colorimetric detection method for 2-AP and EP was developed. The method was successfully used to accurately detect the content of 2-AP in different water samples (tap water, lake water, and seawater) and EP content in Epinephrine Hydrochloride Injection (veterinary). In addition, a cotton-based sensor for rapid field detection of 2-AP in seawater was also fabricated, which provides a reference for the development of low-cost and sensitive sensors. The innovations of this study are as follows: (1) It was discovered for the first time that GHK-Cu has laccase-like activity and it can be obtained without laboratory synthesis. (2) GHK-Cu shows outstanding laccase-like activity and catalytic efficiency (*K*_m_: 0.061 mM, *V*_max_: 1.735 × 10^−4^ mM·s^−1^, *k*_cat_/*K*_m_: 14.3 × 10^–4^ (g·L^–1^)^–1^∙s^–1^). (3) A rapid and accurate colorimetry for EP and 2-AP was developed based on GHK-Cu, and it has been successfully applied to the detection of EP in veterinary injections and 2-AP in real water samples. (4) A cotton-based sensor integrated with a smartphone platform was constructed for the rapid detection of 2-AP in seawater.

## Figures and Tables

**Table 1:** Comparison of the kinetic parameters of different catalysts with laccase-like activity.

Catalyst	[*E*_0_] (mg·mL^−1^)	*K*_m_ (mM)	*V*_max_ (×10^−4^ mM·s^−1^)	*k*_cat_/*K*_m_ (×10^–4^ (g·L^–1^)^−1^∙s^–1^)	Ref.
Laccase	10	0.043	0.083	0.195	This work
GHK-Cu	2	0.061	1.735	14.3	This work
Cu/GMP	0.1	0.590	0.138	0.234	[17]
Ru SA/GFs	5	0.151	0.344	0.456	[33]
Cu-Mel	1	1.296	0.608	0.469	[34]
CN-MOF-818	0.2	4.481	0.628	0.701	[35]
COP-2	1	0.523	2.19	4.19	[36]
Bpy-Cu	1	0.190	0.247	1.30	[37]
Cu_0.5_Mn_0.5_Sn(OH)_6_	0.1	0.320	0.325	10.2	[38]
FF/ICA-Cu	1	0.188	1.31	6.97	[39]
CMC-PtNPs	1.96	0.218	1.13	2.64	[40]
Cu/Zn-ZIF	1	0.090	0.593	6.59	[41]
Co-2MI	1	0.300	0.623	2.08	[42]
Mn-F-Cu	1	0.650	0.663	1.02	[43]
S-FeCo-NC	5	0.150	0.310	0.413	[44]

GMP: Guanosine monophosphate; SA: Single-atom; GFs: 3D porous graphene; Mel: Melamine; MOF: Metal–organic framework; COP: Covalent organic polymers; Bpy: 4,4′-bipyridine; FF: Diphenylalanine peptide; ICA: 4-imidazolecarboxaldehyde; CMC: Carboxymethylcellulose; NPs: Nanoparticle; 2MI: 2-methylimidazole; F: L-phenylalanine.

**Table 2:** Comparison of different methods for the detection of EP.

Material	Methods	Linear Range (μM)	LOD (μM)	Ref.
GHK-Cu	Colorimetry	20–240	9.5	This work
CuSn(OH)_6_	Colorimetry	50–150	14.6	[38]
Cu_0.5_Mn_0.5_Sn(OH)_6_	Colorimetry	20–200	9.3	[38]
Cu_2_O nanospheres	Colorimetry	0–200	10	[47]
CH-Cu	Colorimetry	27.0–270.0	27.0	[48]
Adenine phosphate-Cu	Colorimetry	5.0–200.0	5.0	[49]
Cu-fumarate	Colorimetry	2.7–54.6	2.7	[50]
CTNs	Colorimetry	4.5–90	4.5	[51]
CTAB	DPV	10–270	7.02	[52]
NiO-rGO	Electrochemistry	50–1000	10	[53]
VO_2_ Nanosheets	Fluorimetry	100–1000	48.1	[54]

CH: Cys-His dipeptide; CTNs: Cu-tannic acid nanohybrids; CTAB: Cetyltrimethyl ammonium bromide; DPV: differential pulse voltammetry; rGO: Reduced graphene oxide.

**Table 3:** Colorimetric test results of Epinephrine Hydrochloride Injection (veterinary).

Samples	Concentration (μM)	Found (μM)	Comparison Value (%)	RSD (%) (*n* = 3)
Epinephrine Hydrochloride Injection	227.6	240.5	105.6	1.4
	227.6	246.3	108.2	0.9
	227.6	246.9	108.5	1.1

**Table 4:** Comparison of different methods for the detection of 2-AP.

Material	Method	Linear Range (μM)	LOD (μM)	Ref.
GHK-Cu	Colorimetry	14–100 ^a^	2.56 ^a^	This work
		2–120 ^b^	1.65 ^b^	
Tp-COF@Cu-BDC/GCE	Electrochemistry	2–250	0.597	[55]
Cys-Cu/NH_2_-ZIF-8	Colorimetry	30–300	8.92	[58]
Manganese silicate/GCE	Electrochemistry	0.1–70	18 × 10^−3^	[59]
Au NPs/r-GO/GCE	Electrochemistry	0.4–50	0.093	[60]
Poly-L-arginine-cyclodextrin/CNTs@GNRs/GCE	Electrochemistry	0.025–1.3	6.2 × 10^−3^	[61]
Heterojunction CeO_2_–TiO_2_/GCE	Electrochemistry	0.01–500	3.5	[62]

^a^: results measured in ultrapure water. ^b^: results measured in sea water. Tp: 2,4,6-trihydroxybenzene-1,3,5-tricarbaldehyde; COF: Covalent organic framework; BDC: Terephthalic acid; GCE: Glassy carbon electrode; Cys: Cysteine; CNTs: Carbon nanotubes; GNRs: Graphene nanoribbon.

**Table 5:** Results of 2-AP detection in environmental water samples.

Samples	Added (μM)	Found (μM)	Recovery (%)	RSD (%) (*n* = 3)
Tap Water	20	20.8	104.0	2.1
	40	41.0	102.6	1.8
	60	62.7	104.5	0.8
Jin Lake	20	21.9	109.5	2.0
	40	41.8	104.4	0.6
	60	62.9	104.8	0.4
Bohai Sea	20	18.7	93.5	4.1
	60	62.4	104.0	1.1
	100	101.6	101.6	2.0
Bohai Sea (using the cotton-based sensor to detect)	100	101.4	101.4	3.1
	200	191.2	95.6	1.7
	400	39.8	99.6	0.8

## Data Availability

The original contributions presented in this study are included in the article. Further inquiries can be directed to the corresponding authors.
